# The histone deacetylase SIRT6 promotes glycolysis through the HIF-1α/HK2 signaling axis and induces erlotinib resistance in non-small cell lung cancer

**DOI:** 10.1007/s10495-022-01751-y

**Published:** 2022-08-01

**Authors:** Qiai You, Jianmin Wang, Yongxin Yu, Feng Li, Lingxin Meng, Mingjing Chen, Qiao Yang, Zihan Xu, Jianguo Sun, Wenlei Zhuo, Zhengtang Chen

**Affiliations:** Institute of Cancer, Xinqiao Hospital, Army Medical University, Chongqing, 400037 China

**Keywords:** NSCLC, EGFR-TKI, Erlotinib, SIRT6, Glycolysis

## Abstract

Erlotinib is a first-generation epidermal growth factor receptor tyrosine kinase inhibitor (EGFR-TKI). Overcoming erlotinib resistance is crucial to improve the survival of advanced non-small cell lung cancer (NSCLC) patients with sensitive EGFR mutations. It is also an important clinical problem that urgently needs a solution. In this study, we explored strategies to overcome erlotinib resistance from the perspective of energy metabolism. SIRT6 is a histone deacetylase. Here, we found that high expression of SIRT6 is associated with poor prognosis of lung adenocarcinoma, especially in EGFR-mutated NSCLC patients. The next cell experiment found that SIRT6 expression increased in erlotinib-resistant cells, and SIRT6 expression was negatively correlated with the sensitivity of NSCLC to erlotinib. Inhibition of SIRT6 promoted erlotinib-induced apoptosis in erlotinib-resistant cells, and glycolysis in drug-resistant cells was also inhibited. Functional studies have shown that SIRT6 increases glycolysis through the HIF-1α/HK2 signaling axis in drug-resistant cells and inhibits the sensitivity of NSCLC cells to erlotinib. In addition, the HIF-1α blocker PX478-2HCL attenuated the glycolysis and erlotinib resistance induced by SIRT6. More importantly, we confirmed the antitumor effect of SIRT6 inhibition combined with erlotinib in NSCLC-bearing mice. Our findings indicate that the cancer metabolic pathway regulated by SIRT6 may be a new target for attenuating NSCLC erlotinib resistance and has potential as a biomarker or therapeutic target to improve outcomes in NSCLC patients.

## Introduction

Lung cancer ranks first in mortality among all cancers, and almost one in four people who die from tumors die of lung cancer, of which non-small cell lung cancer (NSCLC) is the most common subtype with an incidence of approximately 85% [1, 2]. Erlotinib, a first-generation epidermal growth factor receptor tyrosine kinase inhibitor (EGFR-TKI), is the first-line treatment for advanced NSCLC patients with EGFR-sensitive mutations (19del or L858R) [3, 4]. However, almost all patients inevitably develop acquired resistance after several months of treatment with erlotinib. Many resistance mechanisms have been discovered, including the EGFR secondary mutation T790M, bypass activation, and epithelial-mesenchymal transition [5, 6]. The abovementioned complex drug resistance mechanisms present challenges for the development of new therapeutic drugs. Therefore, exploring the mechanism of erlotinib resistance in NSCLC and seeking effective strategies to overcome resistance are of great significance to improve the prognosis of patients and to reduce the mortality of lung cancer.

In recent years, metabolism has gradually become a research hotspot in cancer, and increasing evidence shows that cell energy metabolism plays an important role in the occurrence and development of tumors. Energy is needed for all biological activities in organisms; especially in the setting of drug intervention, cells greatly increase their demand for energy to survive. Therefore, the development of drug resistance in tumor cells must be accompanied by an increase in the energy demand [7]. Based on this hypothesis, we started our search for effective strategies to overcome TKI resistance with energy metabolism. Unlike the energy source in normal tissues, the main energy source in tumor cells in both aerobic and anaerobic states is glycolysis, specifically aerobic glycolysis, constituting the "Warburg effect" [8, 9]. Glycolysis not only provides energy for the proliferation of tumor cells but also provides intermediate products (lactic acid and pyruvate), which are important raw materials for the proliferation of tumor cells [10–12]. A previous study found that Cpd64 (a selective PDK inhibitor) enhances the anticancer activity of EGFR-TKI in EGFR-mutant NSCLC cell lines under hypoxic conditions [13]. In addition, 2-doxy-d-glucose (2-DG) is a glucose mimic and an inhibitor of the first rate-limiting step of glycolysis. 2-DG can effectively inhibit the glycolysis process and reduce the volume of afatinib-resistant NSCLC xenografts [14]. These studies show that inhibiting glycolysis might enhance the sensitivity of NSCLC to targeted therapy, and the development of glycolysis inhibitors for antitumor therapy is of great research significance and has broad clinical application prospects.

According to reports in the literature, the hypoxic environment stimulates the production of lactic acid in tumor cells by activating hypoxia-inducible transcription factor 1α (HIF-1α)-dependent genes such as glucose transporter 1 (GLUT1) and hexokinase 2 (HK2) [15–17]. Hypoxia can also induce the expression of monocarboxylate transporter 4 (MCT4), which enhances lactate transport and thus promotes glycolysis [18]. It is known, however, that glycolysis is the primary energy source in tumor cells even under conditions of sufficient oxygen or normoxia, so there must be other important aspects of the regulation of glycolysis.

Interestingly, histone deacetylases (HDACs) can play a critical role in the regulation of glucose metabolism [19]. In addition, HDAC inhibitors have been approved worldwide for the treatment of cutaneous T-cell lymphoma and head and neck cancer due to their antitumor properties reported by clinical studies [20–22]. Therefore, the development of HDAC inhibitors that can regulate the glycolysis level in NSCLC cells and thereby enhance sensitivity to erlotinib attracted our interest.

Sirtuins are mammalian homologues of the yeast histone deacetylase Sir2 and are NAD + -dependent HDACs with seven subtypes, SIRT1-SIRT7. Studies have found that sirtuins can directly interact with genes and enzymes related to glucose metabolism, such as HK2 and lactate dehydrogenase A (LDHA), and/or indirectly regulate the upstream factors or pathways of glycolysis, such as HIF-1/2, to control the level of glycolysis in cancer cells [23]. The SIRT1/HIF1α axis stimulates glycolysis-associated genes and regulates immune responses [24]. The SIRT2/cMYC pathway is involved in the conversion of glucose oxidative metabolism to serine anabolic metabolism in cholangiocarcinoma (CCA) [25]. SIRT3 participates in ATP production by regulating the acetylene and pyruvate dehydrogenase complex (PDH) [26]. SIRT4 inhibits malignant progression of NSCLC through mitochondrial dynamics mediated by the ERK-DRP1 pathway [27]. SIRT5 supports the anaplerotic entry of glutamine into the tricarboxylic-acid (TCA) cycle in malignant phenotypes of colorectal cancer (CRC) via activating glutamate dehydrogenase 1 (GLUD1) [28]. The SIRT7/mH2A1.1 axis contributes to the regulation of the glucose starvation response [29].

In particular, SIRT6 is a core member of the sirtuin family that is involved in regulating various biological processes, such as glycolysis, glutamine metabolism and fatty acid metabolism of tumor cells [30]. SIRT6 has been shown to interact with the transcription factor HIF-1α and is associated with the promoters of a subset of HIF-1α target genes (related to glucose metabolism) in a HIF-1α-dependent manner [31]. In addition, research has shown that overexpression of SIRT6 prevents FOXO3 translocation into the nucleus and doxorubicin-induced cell death [32]. Conversely, SIRT6 depletion increases the sensitivity of melanoma to MAPK inhibition (MAPKi) [33]. Moreover, OSS-128167, a novel SIRT6-specific inhibitor which can decrease cell viability in primary diffuse large B-cell lymphoma (DLBCL), exerts anti-tumor activity in DLBCL cells. It was also reported that OSS-128167 can sensitize primary multiple myeloma (MM) cells, as well as doxorubicin- and melphalan- resistant MM cell lines to chemotherapy [34, 35]. Furthermore, JYQ-42 inhibited the activity of SIRT6 and had a killing effect on pancreatic cancer cells [36], and even a SIRT6 allosteric activator (MDL-800) was reported to have anticancer effects [37]. Given the complexity of SIRT6 functionality, and the anticancer potential of SIRT6 modulators, we sought to determine whether SIRT6 is related to erlotinib resistance and whether the resistance mechanism is related to energy metabolism, especially glycolysis. Here, we aimed to explore the function and mechanism of SIRT6 in regulating the sensitivity of NSCLC cells to erlotinib.

In this study, we used bioinformatics techniques to find that high expression of SIRT6 is associated with poor prognosis of lung adenocarcinoma. Next, SIRT6 expression in PC9, HCC827, PC9/ER and HCC827/ER cells was modified via a lentiviral system. Glycolysis assays, cell apoptosis assays, flow cytometry and western blotting were carried out to analyze the biological function of SIRT6. A nude mouse xenograft model was established for in vivo experiments. Finally, we found that SIRT6 increased glycolysis through the HIF-1α/HK2 signaling axis in drug-resistant cells and inhibited the sensitivity of NSCLC cells to erlotinib. SIRT6 may serve as a prognostic biomarker or therapeutic target for patients with NSCLC.

## Materials and methods

### Analysis of the cancer genome atlas (TCGA) database

ULCAN (http://ualcan.path.uab.edu./analysis.html) [38] is a comprehensive, user-friendly, and interactive web resource for analyzing cancer OMICS data. It is designed to provide easy access to publicly available cancer OMICS data (TCGA, MET500, CPTAC and CBTTC). Through this website, we obtained data from 503 lung squamous carcinoma (LUSC) samples and 52 paired normal samples in the TCGA database, as well as 515 lung adenocarcinoma (LUAD) samples and 59 paired normal samples. Paired t tests were used to compare the expression levels of the SIRT6 genes between normal lung tissues and lung cancer tissues. The Kaplan–Meier plotter (http://kmplot.com/analysis) [39] is capable of assessing the effect of 54 k genes (mRNA, miRNA, protein) on survival in 21 cancer types, including breast (n = 6234), ovarian (n = 2190), lung (n = 3452), and gastric (n = 1440) cancer. Sources for the databases include GEO, EGA, and TCGA. In this study, a lung cancer survival dataset was obtained, which contained 965 low SIRT6 expression cases and 960 high SIRT6 expression cases. We further analyzed the LUAD survival data of 363 low SIRT6 expression cases and 356 high SIRT6 expression cases. Importantly, we downloaded the EGFR-mutant NSCLC patients' survival data in the TCGA database (https://portal.gdc.cancer.gov/), which contained 32 low SIRT6 expression cases and 34 high SIRT6 expression cases, and further analyzed the survival data of EGFR-mutant NSCLC patients.

### Cell culture

PC9 cells were purchased from the Shanghai Institute of Biochemistry and Cell Biology (Shanghai, China), and HCC827 cells were obtained from the American Type Culture Collection (Beijing, China). Both cell lines were exposed to high-dose (1–5 µM) erlotinib (Selleck Chem, Houston, USA) for a short time (72 h), and the surviving cells were continuously exposed to low doses of erlotinib (0.1 µM). This erlotinib administration approach was used for 8 months to establish resistant cell lines, PC9/ER and HCC827/ER, which were maintained by culturing in medium containing 1 µM erlotinib. The two resistant cell lines did not have a secondary T790M mutation, and the identity of all cell lines was confirmed by short tandem repeat (STR) profiling. All cells were maintained in RPMI-1640 medium (HyClone, Utah, USA) supplemented with 10% FBS (HyClone, Utah, USA) and 1% penicillin/streptomycin (Gibco, USA) at 37 °C in a humidified 5% CO_2_ atmosphere.

### Cell viability assay

Cells (5000) were seeded into 96-well plates for 12 h and incubated with erlotinib at concentrations ranging from 0.01 to 100 µM for 48 h. CCK-8 reagent (DOJINDO, Kyushu Island, Japan) was added to each well for a 1 h incubation. The absorbance was measured at 450 nm using a microplate reader (Varioskan Flash, Thermo, USA).

### siRNA transfection

The siRNA against SIRT6 and the control siRNA were purchased from GenePharma (Shanghai, China), and the short-hairpin RNA sequences were si-SIRT6-1: sense, 5ʹ-UCCAUCACGCUGGGUACAUTT-3ʹ and anti-sense, 5ʹ-AUGUACCCAGCGUGAUGGATT-3ʹ; si-SIRT6-2: sense, 5ʹ-GGAAGAAUGUGCCAAGUGUTT-3ʹ and anti-sense, 5ʹ-ACACUUGGCACAUUCUUCCTT-3ʹ; si-NC: sense, 5ʹ-UUCUCCGAACGUGUCACGUTT-3ʹ and anti-sense, 5ʹ-ACGUGACACGUUCGGAGAATT-3ʹ. A total of 2 × 10^5^ cells/well were seeded in 6-well plates and incubated overnight. Then, cultured cells were transiently transfected according to the manufacturer’s instructions, and the final concentration of siRNA was 100 nM (RiboBio, Guangzhou, China).

### Lentiviral transfection of target genes

Our results showed that the si-SIRT6-2 sequence had a stronger interference effect on SIRT6, so we chose this sequence to construct a lentiviral vector and transfect resistant cells. The lentiviral vectors for SIRT6 overexpression and knockdown were purchased from GeneChem (Shanghai, China). A total of 5 × 10^4^ cells/well were seeded in 12-well plates and incubated overnight; then, according to the manufacturer's recommendations, the cultured cells were infected with HitransG and lentivirus (MOI = 100) for 12 h and then cultured in fresh culture medium. After 72 h of infection, flow cytometry was used for detection and sorting to establish the stable cell lines PC9/vector, HCC827/vector, PC9/SIRT6, HCC827/SIRT6, PC9/ER/shCtrl, HCC827/ER/shCtrl, PC9/ER/shSIRT6, and HCC827/ER/shSIRT6.

### Lactate production assay

The same number of cells (3 × 10^5^) was seeded in 6-well plates, and after 48 h of incubation with or without 1 µM erlotinib, lactic acid production was measured using a lactate assay kit according to the manufacturer's instructions (Nanjing Jiancheng, Nanjing, China). After the samples sufficiently reacted with the assay reagents, the absorbance of the mixture in each well was measured at 530 nm using a microplate reader (Varioskan Flash, Thermo, USA), and the lactic acid concentration was calculated. The formula was as follows: lactic acid content (mmol/L) = [(test OD value − blank OD value)/(standard OD value − blank OD value)] × standard concentration (3 mmol/L) × dilution factor before the test.

### Intracellular ATP assay

The same number of cells (3 × 10^5^) was seeded in 6-well plates, and after 48 h of incubation with or without 1 µM erlotinib, the culture medium was centrifuged at 12,000×*g* for 10 min. The supernatant was collected, and lactic acid production was measured using a lactate assay kit according to the manufacturer's instructions (Nanjing Jiancheng, Nanjing, China). After the supernatant samples or standard sufficiently reacted with the assay reagents at 37 °C for 10 min, the absorbance of the mixture in each well was measured at 530 nm using a microplate reader (Varioskan Flash, Thermo, USA), and the lactic acid concentration was calculated. The formula was as follows: lactic acid content (mmol/L) = [(test OD value − blank OD value)/(standard OD value − blank OD value)] × standard concentration (3 mmol/L) × dilution factor before the test.

### RT–qPCR

After total RNA was extracted using RNAiso Plus (TaKaRa, Dalian, China), total RNA (1 µg) with gDNA Eraser was reacted at 42 °C for 2 min. Then, according to the manufacturer's instructions, the reaction product was reverse transcribed at 37 °C for 15 min (PrimeScript™ RT reagent kit, TaKaRa, Dalian, China) to produce cDNA. RT–qPCR was carried out on a CFX384 Touch fluorescence qPCR system (Bio–Rad, USA) with a SYBR Premix Ex Taq (TaKaRa, Dalian, China) kit. The 2^−ΔΔCq^ method was used to calculate relative mRNA expression levels [40]. The primers were as follows: SIRT6, (F) 5ʹ-AGTCTTCCAGTGTGGTGTTCC-3ʹ, (R) 5ʹ-CAAAGGTGGTGTCGAACTTGG-3′; HIF-1α, (F) 5ʹ-ATCCATGTGACCATGAGGAAATG-3ʹ, (R) 5ʹ-TCGGCTAGTTAGGGTACACTTC-3ʹ; HK2, (F) 5ʹ-GAGCCACCACTCACCCTACT-3ʹ, (R) 5ʹ-CCAGGCATTCGGCAATGTG-3ʹ; and β-actin, (F) 5ʹ-GCGAGCACAGAGCCTCGCCTT-3ʹ; (R) 5ʹ-CATCATCCATGGTGAGCTGGCGG-3ʹ. The mRNA expression levels were normalized to those of β-actin.

### Western blot analysis

The cells were washed three times with PBS, and protein lysate buffer containing 5 µL PMSF (1 mM) per 500 µL was placed on ice for 5 min and centrifuged at 14,000×*g* for 10 min after sonication. Then, the supernatant was collected. The protein concentration was measured using a bicinchoninic acid (BCA) protein assay kit (Beyotime, Shanghai, China). After boiling with loading buffer for 10 min, equal amounts of protein (40 µg) were separated by 10% SDS–PAGE (Beyotime, Shanghai, China) and transferred onto PVDF membranes (Millipore, Massachusetts, USA). Membranes were blocked with 5% milk powder for 60 min at room temperature and incubated with specific primary antibodies at 4 °C overnight. Rabbit anti-SIRT6 (1:1000 dilution, Abcam, UK), rabbit anti-HIF-1α (1:500 dilution, Abcam, UK), rabbit anti-HK2 (1:500 dilution, Cell Signaling Technology, USA), and mouse anti-β-actin (1:1000 dilution, ZSGB-Bio, Beijing, China) antibodies were used. Proteins were detected using anti-rabbit IgG (1:2000 dilution, Cell Signaling Technology, USA) or anti-mouse IgG (1:2000 dilution, Cell Signaling Technology, USA) antibodies and a chemiluminescence detection system (FluorChem HD2, USA).

### Flow cytometric analysis of apoptosis

For the apoptosis analysis, cells were incubated with 1 µM erlotinib for 48 h, harvested, and washed with cold PBS. Then, 5 µL PE Annexin V and 5 µL 7-AAD Viability Staining Solution were added to 100 µL cell suspension. After staining at room temperature (25 °C) for 15 min in the dark, 400 µL of Annexin V Binding Buffer (all from BioLegend, USA) was added to stop the reaction, and the cells were analyzed using flow cytometry (Gallios, Beckman, USA).

### Xenograft studies

Female nude mice aged 4 to 6 weeks were obtained from the SPF laboratory animal center of Xinqiao Hospital (Chongqing, China). All animal experiments were approved by the Institutional Animal Care and Use Committee of Xinqiao Hospital, and all procedures involving mice were performed according to the animal care guidelines of Xinqiao Hospital of Army Medical University. Normal saline (100 µL) containing 1 × 10^6^ cells was injected into the right axilla of the mice to establish xenograft tumors, and the mice were treated with erlotinib at 60 mg/kg by gavage for 14 consecutive days after 2 weeks. Tumor volume was measured every 3 days and calculated according to the formula V = (length × width 2)/2. After the mice were euthanized by cervical dislocation, tumor tissues were removed and fixed with 4% paraformaldehyde.

### Immunohistochemistry and TUNEL staining

The tumor tissue was fixed with 4% paraformaldehyde for 24 h, embedded in paraffin, and sectioned (slice thickness was approximately 2.5 µm). Tissue sections were dewaxed in xylene and a series of gradient concentrations of ethanol. Dewaxed sections were treated with 3% H_2_O_2_ to remove endogenous catalase, subjected to antigen retrieval in citrate buffer, washed with PBS, blocked with serum, and then incubated overnight at 4 °C with primary antibody. Rabbit anti-SIRT6 (1:200 dilution, Cell Signaling Technology, USA), rabbit anti-HIF-1α (1:100 dilution, Abcam, UK), rabbit anti-HK2 (1:200 dilution, Cell Signaling Technology, USA), and mouse anti-Ki-67 (1:200 dilution, Cell Signaling Technology, USA) antibodies were used. The biotinylated secondary antibody was incubated at room temperature for 1 h and then incubated with horseradish peroxidase-conjugated streptavidin for 30 min, DAB immunostaining for 3 min, and hematoxylin counterstaining of the cells.

TUNEL staining of deparaffinized tissues was performed following the instructions of the In Situ Cell Death Detection Kit (Roche, USA) for 1 h at 37 °C. After terminating the reaction, the cells were washed with PBS 3 times (5 min each time), stained with DAB for 3 min, and counterstained with methyl green for 10 min at room temperature. Then, the cells were dehydrated, mounted, and observed under an optical microscope.

The area and intensity of the positive staining field of the tumor tissues were automatically calculated by Image-Pro Plus software (version 6.0).

### Statistical analysis

All in vitro experimental data were obtained from three or more independent experiments, and values are presented as the mean ± SEM. Statistical analyzes were performed using the GraphPad Prism software package (version 8.0). To determine the difference in mRNA expression level, IC50 value, apoptosis, lactate, ATP and the mean density between the two groups, Student’s t test was performed to determine statistically significant differences, and p < 0.05 indicated that the difference was statistically significant (*).

## Results

### SIRT6 expression is upregulated in NSCLC tissues, and SIRT6 upregulation is associated with poor prognosis in NSCLC patients

**Fig. 1:**
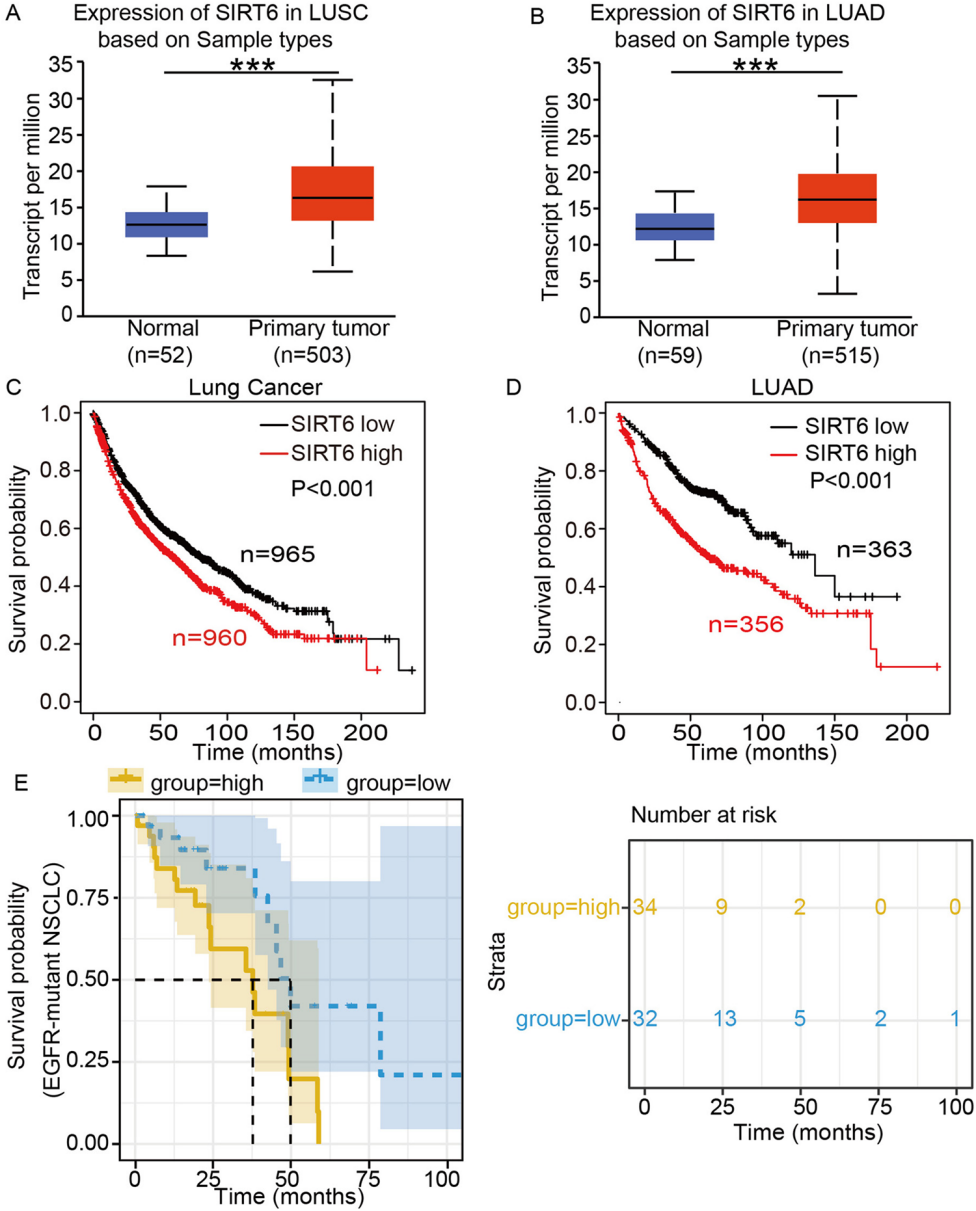
SIRT6 expression is upregulated in NSCLC tissues, and SIRT6 upregulation is associated with poor prognosis in NSCLC patients. SIRT6 is overexpressed in LUSC (**A**) and LUAD (**B**) tissues in comparison with normal lung tissues. mRNA expression data were obtained using UALCAN. Survival analysis of the association of SIRT6 with OS in lung cancer (**C**) and LUAD (**D**) patients from the Kaplan–Meier Plotter website. **E** NSCLC tissue sample information downloaded from the TCGA database. Mutation data, RNA-seq data and clinical information data of samples were integrated to obtain EGFR mutation data of NSCLC, and then the correlation between SIRT6 expression level and overall survival was analyzed. ***p < 0.001

SIRT6, a member of the sirtuin family, is closely related to tumorigenesis and tumor development; however, whether SIRT6 promotes or inhibits tumor growth remains controversial. Recent evidence shows that SIRT6 exerts a tumor-suppressive effect by inhibiting the proliferation and promoting the apoptosis of various cancer cells, such as colon cancer, pancreatic cancer and glioma cells [41–43]. However, other studies have shown that SIRT6 is highly expressed in skin cancers, breast cancers and prostate cancers and is associated with poor prognosis [44–46]. Therefore, to understand the impact of SIRT6 on the prognosis of lung cancer patients, we used public databases to obtain survival data of NSCLC patients. Bioinformatics analysis using UALCAN indicated that SIRT6 expression was upregulated in lung cancer tissues compared to normal lung tissues (sample number in LUSC group: normal 52, primary tumor 503, *p* < 0.001; Fig. 1A) (sample number in LUAD group: normal 59, primary tumor 515, *p* < 0.001; Fig. 1B). To further explore the relationship between SIRT6 and prognosis in lung cancer patients, Kaplan–Meier Plotter was used to analyze survival data, and the results showed that SIRT6 upregulation was significantly associated with poor overall survival in patients with lung cancer (mOS: 59.11 months vs*.* 80.03 months, *p* < 0.001; Fig. 1C), especially in lung adenocarcinoma (mOS: 63.4 months vs*.* 136.33 months, p < 0.001; Fig. 1D). This finding suggests that the high expression of SIRT6 may be a poor prognostic factor for lung cancer patients, especially lung adenocarcinoma patients.

Given that EGFR-sensitive mutations account for more than half of lung adenocarcinomas, we next investigated whether SIRT6 is also involved in the TKI resistance mechanism associated with poor prognosis in these patients. We analyzed the data of EGFR-mutated NSCLC patients from TCGA and found that the SIRT6 expression level was negatively correlated with OS. Compared with the SIRT6 high expression group, the OS with low SIRT6 expression group was significantly prolonged (mOS: 37.5 months vs*.* 50 months, *p* < 0.001; Fig. 1E). The above results suggested that SIRT6 may be related to EGFR-TKI resistance, so we performed a series of experiments to explore its possible mechanisms.

### SIRT6 is highly expressed in erlotinib-resistant cells

**Fig. 2:**
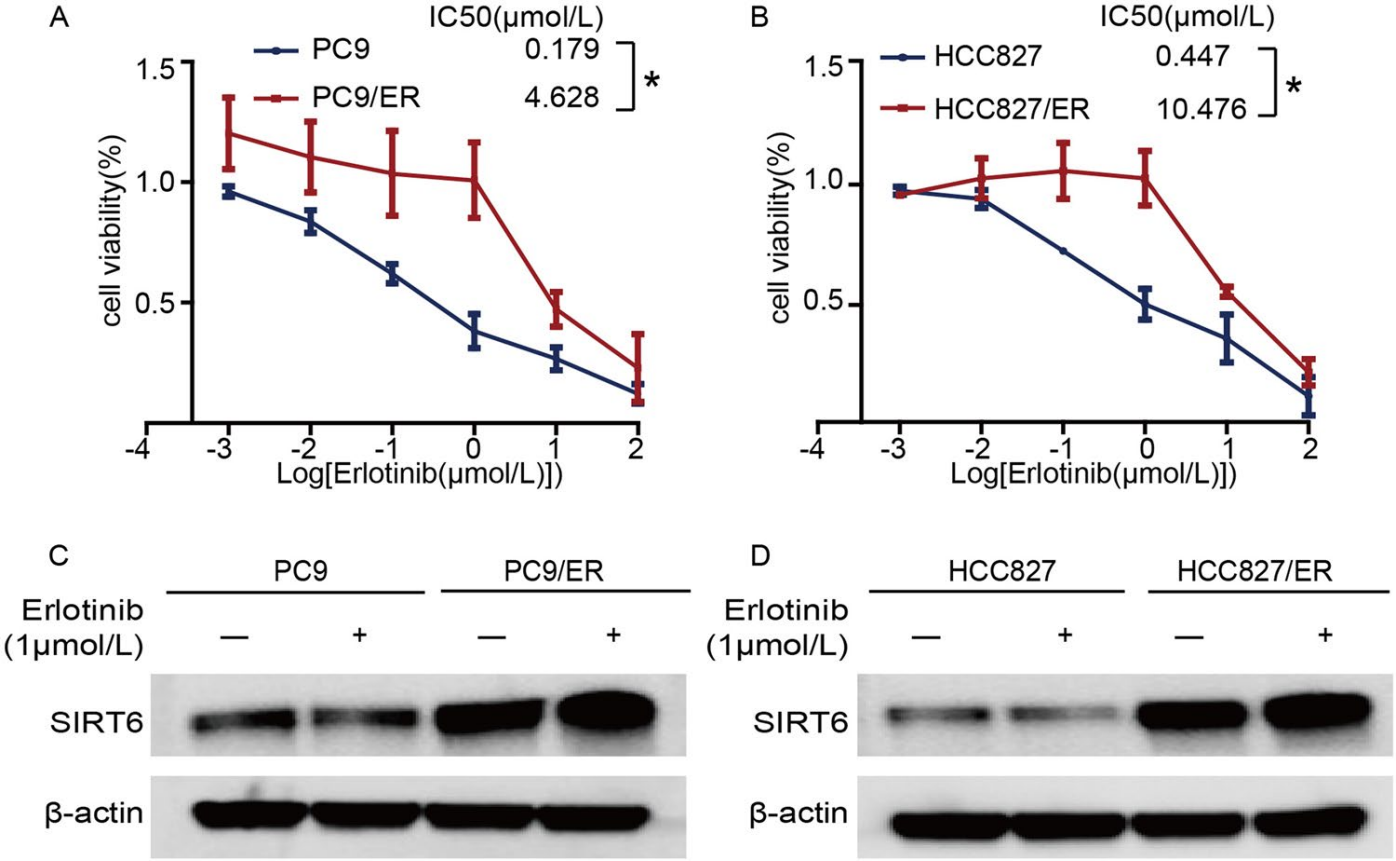
SIRT6 is highly expressed in erlotinib-resistant cells. **A** and **B** The sensitivity of PC9, PC9/ER, HCC827, and HCC827/ER NSCLC cells to erlotinib was detected using a CCK-8 kit after treatment or not with erlotinib for 48 h. **C** and **D** PC9, PC9/ER, HCC827, and HCC827/ER NSCLC cells were treated or not with 1 µM erlotinib for 48 h. Cell lysates were prepared and subjected to western blot analysis using the indicated antibodies. *p < 0.05, **p < 0.01

An approach combining high-dose shock (1–5 µM erlotinib) and low-dose maintenance (0.01 µM erlotinib) was used to establish cells with sustained erlotinib drug resistance, namely, PC9/ER and HCC827/ER cells, and neither of these cell lines harbored the T790M mutation [47]. Consistent with our expectations, the IC50 value of erlotinib in resistant cells was significantly higher than that in sensitive cells (the IC50 values in PC9 vs*.* PC9/ER cells were 0.179 µM vs*.* 4.628 µM, *p* < 0.05) (Fig. 2A) (the IC50 values in HCC827 vs. HCC827/ER cells were 0.447 µM vs*.* 10.476 µM, *p* < 0.05) (Fig. 2B), indicating that we can use these two cell lines for subsequent studies on the mechanism of erlotinib resistance.

We performed immunoblotting experiments and found that SIRT6 protein expression in PC9/ER and HCC827/ER cells was higher than that in the corresponding parent cells. More interestingly, after 48 h of treatment with 1 µM erlotinib, the expression of SIRT6 in drug-resistant cells was still higher than that in drug-sensitive cells (Fig. 2C, D). These data demonstrate that SIRT6 is highly expressed in drug-resistant cells and cannot be inhibited by erlotinib, which suggests that SIRT6 may be related to erlotinib resistance in NSCLC.

### Inhibition of SIRT6 attenuates the resistance of NSCLC cells to erlotinib and enhances the cell apoptosis induced by erlotinib

**Fig. 3:**
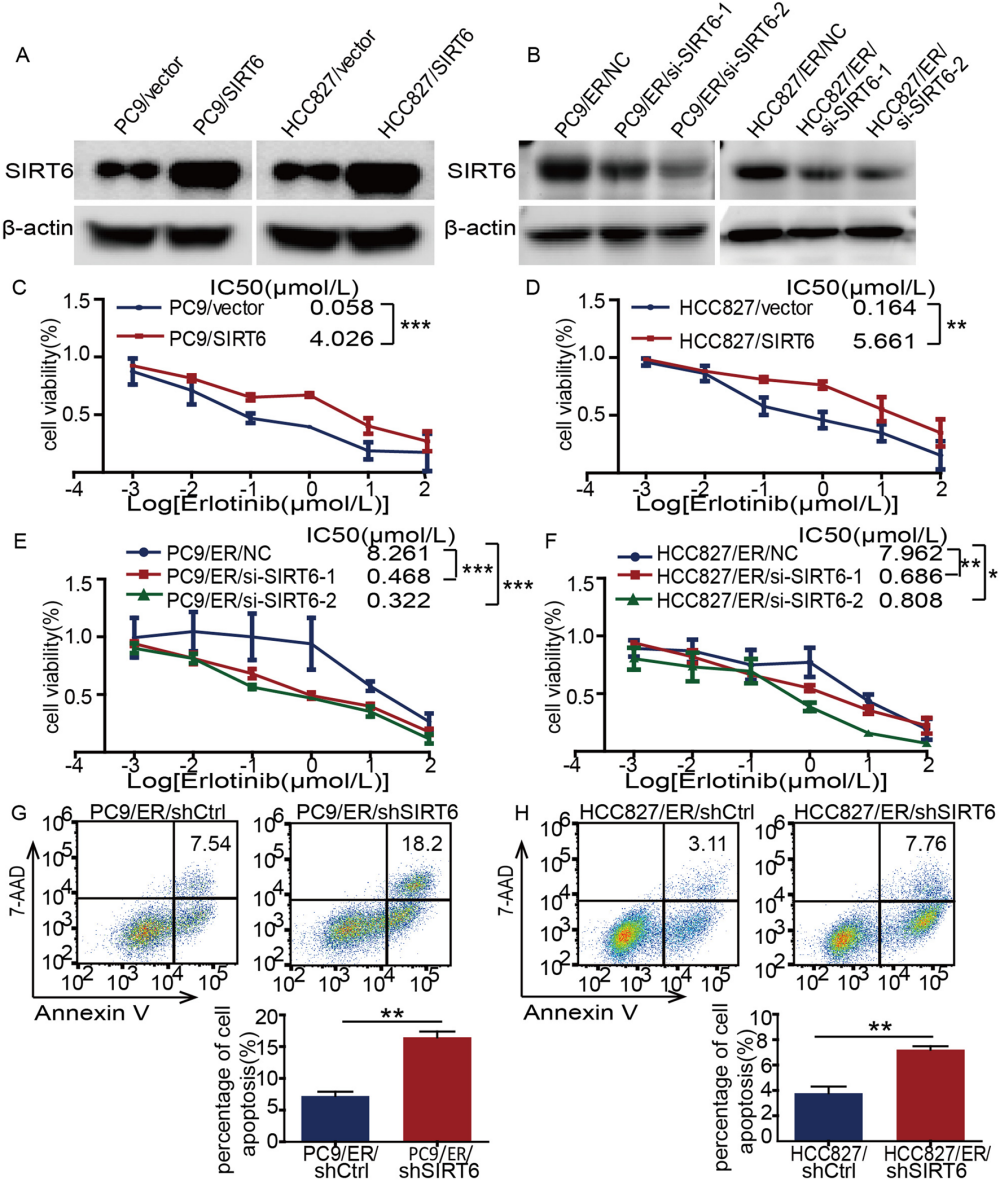
Inhibition of SIRT6 attenuates the resistance of NSCLC cells to erlotinib and enhances the cell apoptosis induced by erlotinib. **A** The PC9/vector, HCC827/vector, PC9/SIRT6, and HCC827/SIRT6 cell lines were established using PC9 and HCC827 cells transfected with SIRT6-overexpressing vector and control vector. SIRT6 protein expression was analyzed using western blotting. **B** After transfecting si-NC, si-SIRT6-1 and si-SIRT6-2 in erlotinib-resistant cell lines (PC9/ER and HCC827/ER), the cell lines PC9/ER/si-SIRT6-1, PC9/ER/si-SIRT6-2 and HCC827/ER/si-SIRT6-1, HCC827/ER/si-SIRT6-2 and the corresponding control cell lines PC9/ER/NC and HCC827/ER/NC were established, and the protein levels of SIRT6 were analyzed using western blotting. The overexpression of SIRT6 in PC9 and HCC827 cells (**C**, **D**) and the influence of SIRT6 knockdown (**E**, **F**) on erlotinib sensitivity in PC9/ER and HCC827/ER cells were measured using the CCK-8 assay. **G**, **H** SIRT6-knockdown cells were treated with 1 µM erlotinib for 48 h, and erlotinib-mediated apoptosis was detected using flow cytometry. *p < 0.05, **p < 0.01, ***p < 0.001

To determine whether SIRT6 is related to erlotinib resistance in NSCLC, we first constructed SIRT6 overexpression using lentiviral transfection technology. PC9/SIRT6 and HCC827/SIRT6 cells were generated by transfecting sensitive parental cell lines with SIRT6 cDNA (Fig. 3A); si-SIRT6-1 and si-SIRT6-2 were transfected into PC9/ER and HCC827/ER cells to construct SIRT6-knockdown cell lines (Fig. 3B). We first determined the effect of SIRT6 on NSCLC cell drug resistance by the CCK-8 method and found that overexpression of SIRT6 could induce the development of erlotinib resistance in cells (the IC50 values in PC9/vector vs*.* PC9/SIRT6 cells were 0.058 µM vs*.* 4.026 µM, *p* < 0.001) (Fig. 3C) (the IC50 values in HCC827/vector vs*.* HCC827/SIRT6 cells were 0.164 µM vs*.* 5.661 µM, *p* < 0.01) (Fig. 3D). Conversely, knockdown of SIRT6 reversed cell resistance to erlotinib (the IC50 values in PC9/ER/NC vs*.* PC9/ER/si-SIRT6-1 cells were 8.261 µM vs. 0.468 µM, *p* < 0.001; the IC50 values in PC9/ER/NC vs. PC9/ER/si-SIRT6-2 cells were 8.261 µM vs*.* 0.322 µM, *p* < 0.001) (Fig. 3E); (the IC50 values in HCC827/ER/NC vs*.* HCC827/ER/si-SIRT6-1 cells were 7.962 µM vs*.* 0.686 µM, *p* < 0.01; the IC50 values in HCC827/ER/NC vs*.* HCC827/ER/si-SIRT6-2 cells were 7.962 µM vs*.* 0.808 µM, *p* < 0.05) (Fig. 3F).

As shown in Fig. 3B, the si-SIRT6-2 sequence had a stronger interference effect on SIRT6, so we chose this sequence to construct a lentiviral vector and transfected resistant cells, and the obtained SIRT6 knockdown cells (PC9/ER/shSIRT6 and HCC827/ER/shSIRT6) were used for subsequent mechanistic exploration.

Next, we evaluated the effect of SIRT6 on the apoptosis of NSCLC cells and found that SIRT6 knockdown promoted apoptosis induced by 1 μM erlotinib (Fig. 3G, H). Collectively, these results show that inhibition of SIRT6 attenuates the resistance of NSCLC cells to erlotinib and enhances NSCLC cell apoptosis when incubated with erlotinib.

### Erlotinib-resistant NSCLC cells have a stronger ability to produce ATP and lactic acid

**Fig. 4:**
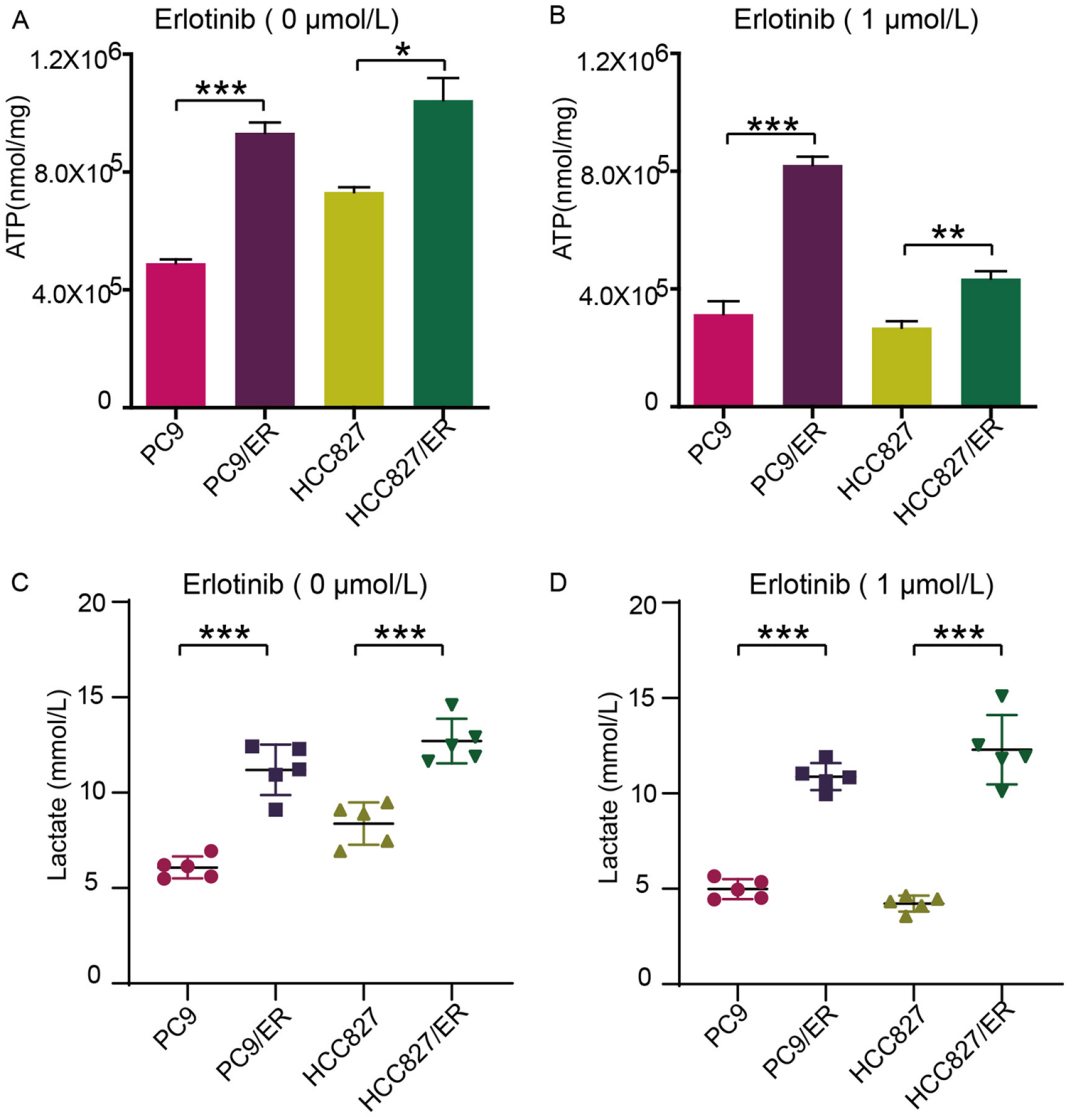
Erlotinib-resistant NSCLC cells have a stronger ability to produce ATP and lactic acid. **A** Intracellular ATP contents of PC9, HCC827, PC9/ER, and HCC827/ER cells were measured. **B** After PC9, HCC827, PC9/ER, and HCC827/ER cells were treated with 1 µM erlotinib for 48 h, the intracellular ATP content was measured. **C** The lactate production of PC9, HCC827, PC9/ER, and HCC827/ER cells was measured. **D** After PC9, HCC827, PC9/ER, and HCC827/ER cells were treated with 1 µM erlotinib for 48 h, lactate production was tested. *p < 0.05, **p < 0.01, ***p < 0.001

The literature indicates that EGFR-TKI treatment can facilitate aerobic glycolysis [13, 48], indicating that NSCLC cells rely more on glycolysis for energy under the action of EGFR-TKIs. Therefore, we hypothesized that SIRT6 regulates glycolysis to promote erlotinib resistance in NSCLC. First, we compared the production of ATP between erlotinib-sensitive and erlotinib-resistant cells, and the data showed that erlotinib-resistant cells produced more ATP (Fig. 4A) than erlotinib-sensitive cells under the same culture conditions, and the same results were seen in the 1 µM erlotinib group (Fig. 4B). Next, we explored whether erlotinib-resistant cells produced more ATP through the TCA pathway or the glycolysis pathway. Consistent with ATP production, regardless of whether 1 µM erlotinib was used for intervention, erlotinib-resistant cells produced more lactic acid (Fig. 4C, D) than sensitive cells under the same culture conditions. This suggests that erlotinib-resistant cells rely more on glycolysis to produce ATP, which is consistent with our previous hypothesis that drug-resistant cells have higher glycolysis levels.

### SIRT6 overexpression can enhance glycolysis in erlotinib-sensitive NSCLC cells, while SIRT6 knockdown attenuates glycolysis in erlotinib-resistant NSCLC cells

**Fig. 5:**
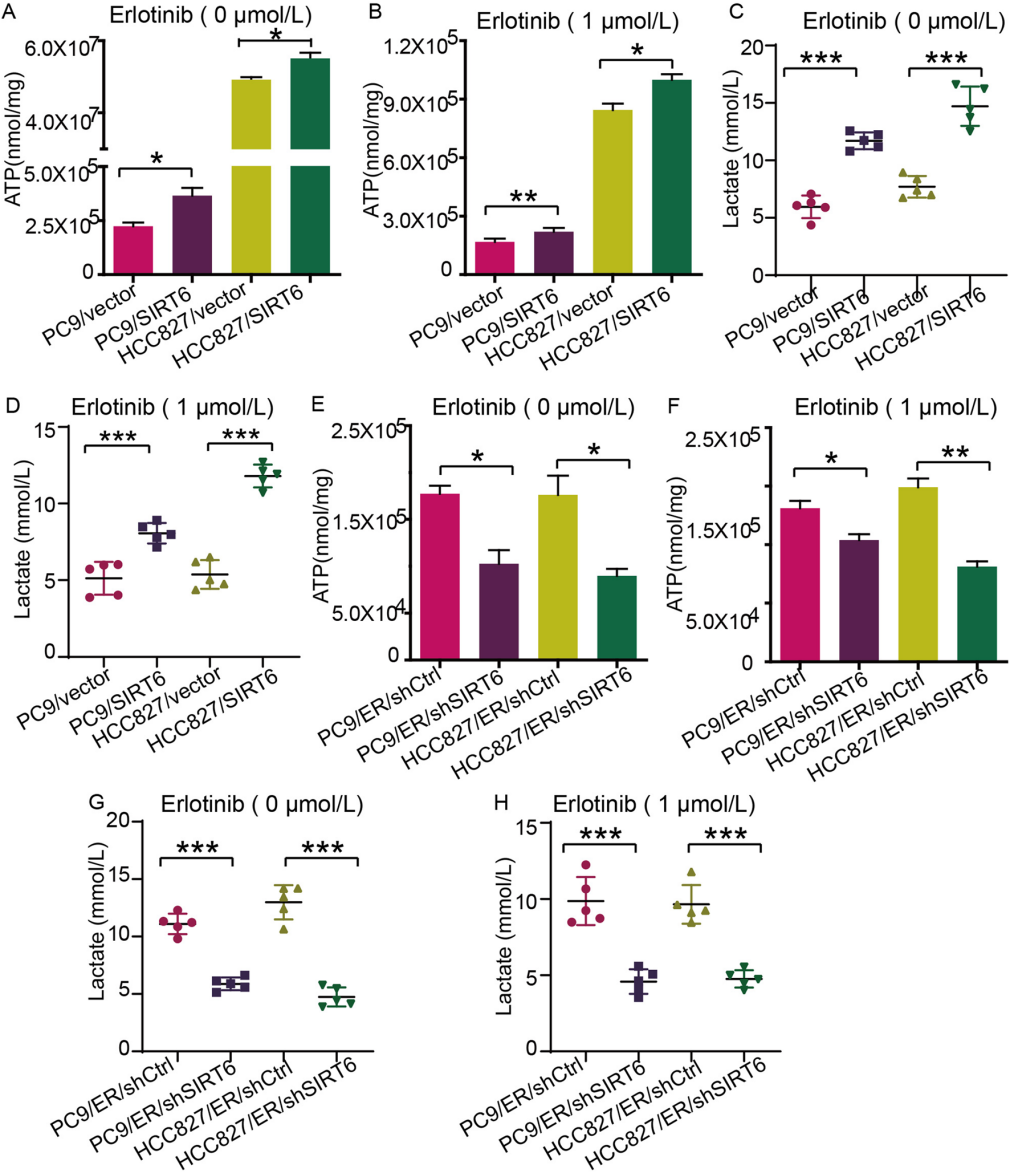
SIRT6 overexpression can enhance glycolysis in erlotinib-sensitive NSCLC cells, while SIRT6 knockdown attenuates glycolysis in erlotinib-resistant NSCLC cells. **A** The ATP content between SIRT6-overexpressing cells and the respective control cells was compared. **B** After treatment of SIRT6-overexpressing cells and the respective control cells with 1 µM erlotinib for 48 h, the ATP content was compared. **C** The lactic acid between SIRT6-overexpressing cells and the respective control cells was compared. **D** After treatment of SIRT6-overexpressing cells and the respective control cells with 1 µM erlotinib for 48 h, the lactic acid was compared. The same method was used to measure the intracellular ATP content (**E, F**) and lactic acid production (**G, H**) of the SIRT6-knockdown cell line and the corresponding control cell line. *p < 0.05, **p < 0.01, ***p < 0.001

Therefore, we next analyzed the influence of SIRT6 on the glycolytic ability of NSCLC cells. As expected, under the same culture conditions, SIRT6-overexpressing NSCLC cells produced more ATP than control cells (Fig. 5A), and when treated with 1 µM erlotinib, drug-resistant cells still had a stronger ability to produce ATP (Fig. 5B), and the lactate production results showed the same trend (Fig. 5C, D). SIRT6-knockdown cells produced less ATP than control cells (Fig. 5E, F). Consistent with the ATP results, the SIRT6-knockdown group produced significantly less lactate than the corresponding control group (Fig. 5G, H). Combined with the results of previous studies, these findings show that SIRT6 can induce erlotinib resistance by promoting glycolysis in NSCLC cells, but its specific regulatory mechanism needs to be further studied.

### SIRT6 promotes glycolysis through the HIF-1α/HK2 signaling pathway to induce erlotinib resistance

**Fig. 6:**
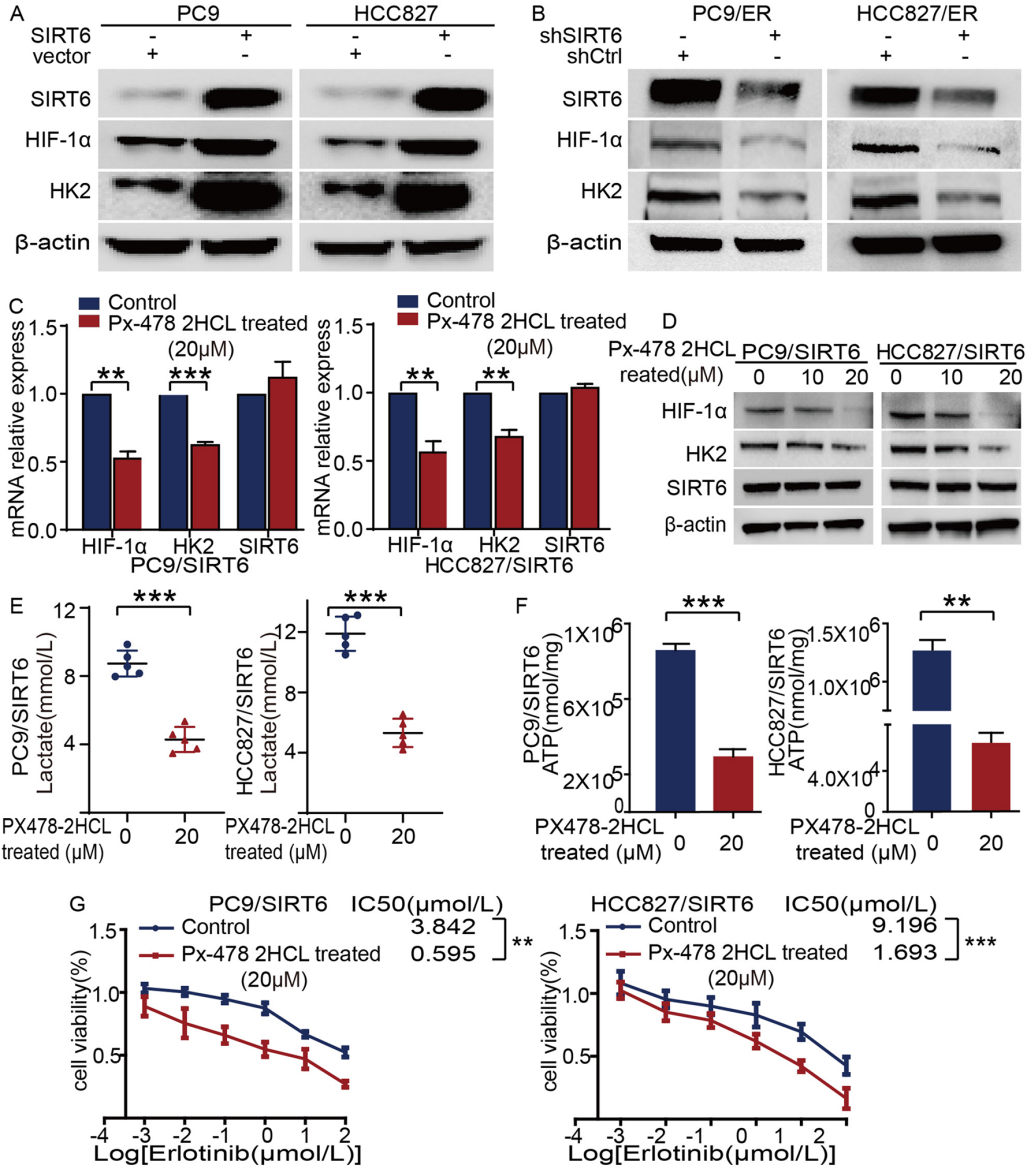
SIRT6 promotes glycolysis through the HIF-1α/HK2 signaling pathway to induce erlotinib resistance. **A**, **B** western blot showing the molecular changes in the SIRT6/HIF-1α/HK2 signaling pathway after overexpression or knockdown of SIRT6 in cells. **C** Changes in the gene levels of related molecules on the signal axis after treatment of PC9/SIRT6 and HCC827/SIRT6 cells with a HIF-1α inhibitor (PX-478 2HCl (Selleck Chem, Houston, USA)) at 20 µM for 48 h. **D** After treatment with 10 µM or 20 µM PX-478 2HCl for 48 h, the cell lysate was used for western blotting to analyze the changes in related molecules. After incubating PC9/SIRT6 and HCC827/SIRT6 cells with or without 20 µM PX-478 2HCl for 48 h, lactic acid production (**E**) and intracellular ATP content (**F**) were compared. After treatment with 20 µM PX-478 2HCl for 48 h in PC9/SIRT6 and HCC827/SIRT6 cells, the influence of the HIF-1α inhibitor on erlotinib sensitivity in SIRT6-overexpressing cells was evaluated using a CCK-8 assay (**G**). *p < 0.05, **p < 0.01, ***p < 0.001

The key function of SIRT6 in regulating aerobic glycolysis has been reported in many tumors. However, to the best of our knowledge, the glycolytic function regulated by SIRT6 has never been explored in NSCLC, especially EGFR-TKI-resistant NSCLC. Our study found that after SIRT6 was overexpressed in erlotinib-sensitive NSCLC cells, the levels of HIF-1α and the glycolytic rate-limiting enzyme HK2 also increased (Fig. 6A). Conversely, HIF-1α and HK2 protein levels were reduced when SIRT6 was knocked down in erlotinib-resistant NSCLC cells (Fig. 6B). Next, to further verify whether SIRT6 regulates glycolysis in NSCLC cells through the HIF-1α/HK2 signaling axis, PX-478 2HCl, a HIF-1α inhibitor, was used to suppress the expression of HIF-1α in SIRT6-overexpressing cells. The results showed that after HIF-1α was successfully inhibited, the expression of HK2 was also decreased, while the expression of SIRT6 did not change significantly (Fig. 6C, D). Interestingly, inhibition of HIF-1α reduced the production of lactic acid (Fig. 6E) and ATP (Fig. 6F) in SIRT6-overexpressing cells. More importantly, HIF-1α inhibitors attenuated the resistance induced by SIRT6 overexpression (in PC9/SIRT6 cells, the IC50 values in control *vs.* PX-478 2HCl (20 µM)-treated cells were 3.842 µM vs. 0.595 µM, *p* < 0.01; in HCC827/SIRT6 cells, the IC50 values in control *vs.* PX-478 2HCl (20 µM)-treated cells were 9.196 µM vs. 1.693 µM, *p* < 0.001) (Fig. 6G). The above results indicate that SIRT6 regulates glycolysis via the HIF-1α/HK2 signaling pathway and further promotes the development of erlotinib resistance in NSCLC cells.

### SIRT6 induces erlotinib resistance in vivo

**Fig. 7:**
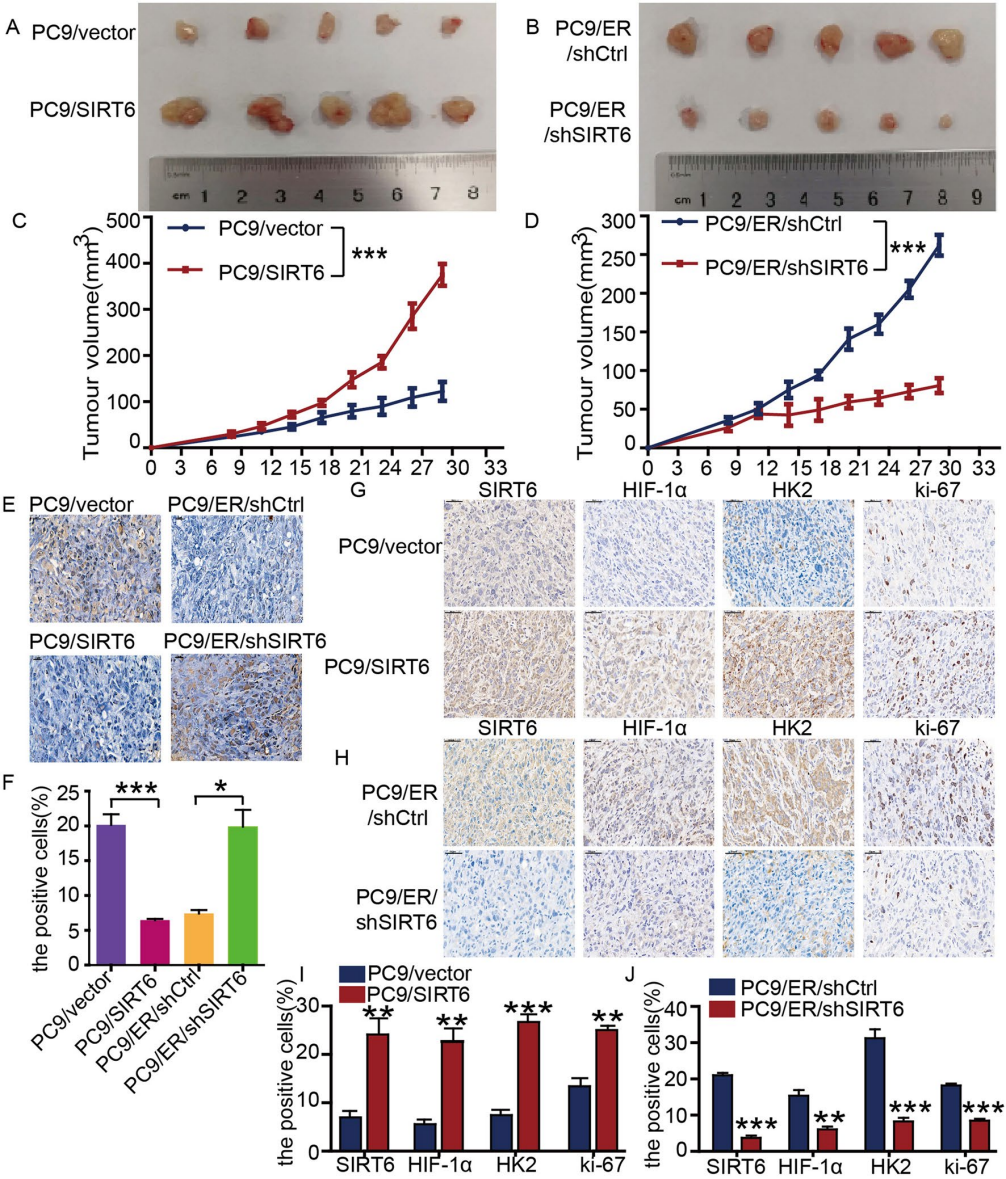
SIRT6 induces erlotinib resistance in vivo. **A**, **B** Two weeks after the establishment of the xenograft model, erlotinib was continuously administered for 14 days, the mice were euthanized, and the tumors were removed (n = 5 mice/group). **C**, **D** Tumor growth curves representing the mean ± SEM of tumor volumes of five mice in the indicated xenograft group. **E** A TUNEL assay (scale bars, 20 µm) was used to analyze cell apoptosis in tumor tissues. **F** The proportion of apoptotic cells in tumor tissues was measured by Image-Pro Plus software. **G**, **H** Immunohistochemical staining (scale bars, 50 µm) to confirm SIRT6, HIF-1α and HK2 protein expression in the designated tumor sample group and Ki-67 staining to measure tumor cell proliferation. **I**, **J** The percentage of SIRT6-, HIF-1α-, HK2-, and Ki-67-positive cells in different groups was analyzed with Image-Pro Plus. *p < 0.05, **p < 0.01, ***p < 0.001

In vivo tumorigenic experiments were conducted to further assess the effect of SIRT6 on the antitumor activity of erlotinib. Stable SIRT6-overexpressing cells (PC9/SIRT6) were injected subcutaneously into nude mice, and the mice were treated with erlotinib. Compared to the control group, the PC9/SIRT6 group showed significantly higher tumor volumes (Fig. 7A, C). Mice injected with stable SIRT6-deficient cells (PC9/ER/shSIRT6) showed smaller tumor volumes than cells injected with PC9/ER/shCtrl (Fig. 7B, D). We further performed TUNEL staining to analyze the apoptosis of tumor tissues in each group and found that the proportion of apoptosis in the SIRT6 overexpression group decreased, while the proportion of apoptosis in the SIRT6 interference group increased (Fig. 7E), and the difference in the proportion of positive cells in each group was statistically significant (Fig. 7F), which is consistent with the results of the in vitro experiment. In addition, immunohistochemical analysis showed that the expression of SIRT6, HIF-1α, HK2 and Ki-67 in tumor tissues of the PC9/SIRT6 group was higher than that of the PC9/vector group (Fig. 7G). Consistent with the tumor volume data, SIRT6, HIF-1α, HK2 and Ki-67 protein levels were lower in the PC9/ER/shSIRT6 tumor tissue samples than in the PC9/ER/shCtrl tissue samples (Fig. 7H). The difference in the expression of SIRT6, HIF-1α, HK2, and Ki-67 in each group was statistically significant (Fig. 7I, J). These results indicate that SIRT6 is essential for inducing erlotinib resistance in vivo. SIRT6 increases the content of HIF-1α and HK2 in cells and enhances resistance to apoptosis induced by erlotinib.

## Discussion

EGFR-TKIs are the first-line drugs for advanced NSCLC patients with EGFR-sensitive mutations. Compared with radiotherapy and chemotherapy, EGFR-TKI targeted therapy exerts better clinical effects, but the disease eventually progresses due to the presence of drug resistance. Therefore, finding strategies to overcome TKI resistance is critical to improving patient survival. The conversion of aerobic respiration to anaerobic glycolysis of cancer cells has been accepted as one of the hallmarks of the occurrence and development of acquired drug resistance [49]. In the present study, we found that high expression of SIRT6 is associated with poor prognosis of lung adenocarcinoma. Furthermore, SIRT6 promotes glycolysis in erlotinib-resistant NSCLC cells through the HIF-1α/HK2 signaling axis, which is the mechanism of drug resistance. More importantly, SIRT6 inhibition and erlotinib treatment synergistically suppress NSCLC xenograft growth in vivo.

SIRT6, one of the core members of the sirtuin family, is considered to act as a double-edged sword in cancer, with dual roles as a tumor suppressor and oncogene [50]. We first used online databases and found that SIRT6 is upregulated in lung cancer, and the overall survival of lung cancer patients with high SIRT6 expression is shorter, suggesting that SIRT6 is associated with poor prognosis of lung cancer, especially EGFR mutant NSCLC. These data corroborate the idea that SIRT6 can also act as an oncogene in NSCLC.

Thus, we aimed to determine whether SIRT6 can induce erlotinib resistance in NSCLC and to identify the specific mechanism in this study. Literature analysis showed that SIRT6 participates in the regulation of various biological processes and plays a key role in the homeostasis of organisms, including the regulation of cell metabolism, DNA repair, and gene expression [51]. SIRT6 deacetylates Beclin-1 in hepatocellular carcinoma (HCC) cells and stimulates the autophagic degradation of E-cadherin, which in turn promotes epithelial-mesenchymal transition (EMT) in HCC cells and ultimately increases metastasis in liver cancer [52]. SIRT6 can mediate the transcriptional regulation of IGFBP2 and therefore activate IGF-1R/AKT, which promotes the development of MAPK inhibitor resistance in melanoma [53]. Downregulation of SIRT6 leads to an increase in doxorubicin-induced death of hepatocellular carcinoma cells by inducing FOXO3 to translocate into the nucleus and bind its target genes P27 and Bim [33]. Among the reports in lung cancer, SIRT6 can regulate EMT and invasion of NSCLC cells [54]. In addition, high expression of SIRT6 is associated with poor prognosis in NSCLC patients, and SIRT6 knockdown increases the sensitivity of lung adenocarcinoma cell lines to paclitaxel [55]. We predicted that the occurrence of erlotinib resistance in NSCLC may also be related to SIRT6, and we conducted related studies to test this hypothesis. Here, we confirmed for the first time that SIRT6 induces erlotinib resistance in NSCLC and that inhibition of SIRT6 can promote erlotinib-induced apoptosis.

Next, we discussed the specific mechanism by which SIRT6 induces erlotinib resistance. A literature search indicated that the Warburg effect is one of the earliest markers of changes in tumor metabolism, and accumulating data suggest that aerobic glycolysis involves numerous molecular and functional processes that support cancer progression [56]. Our group found that under the same culture conditions, erlotinib-resistant NSCLC cells were able to produce more lactic acid and ATP than sensitive cells, revealing that drug-resistant cells tend to rely on glycolysis for energy. Enhanced glycolysis can participate in resistance through multiple mechanisms. By the way, cancer cells can produce a large amount of lactic acid, leading to acidification of the tumor microenvironment. Microenvironmental acidosis is a key factor in the development and malignant progression of cancer, which provides strong evolutionary selection pressure and promotes the emergence of aggressive and antitherapeutic cloning [57]. Moreover, acidified tumor microenvironment can induce genomic instability and mutation of tumor cells, and then show drug resistance [58]. Glycolysis produces a large amount of ATP for drug-resistant cells to synthesize biological macromolecules, and intermediate metabolites in the glycolysis pathway are also used in the proliferation of drug-resistant cells to drive tumor growth. In addition, glycolysis can lead to drug resistance in cells by promoting autophagy [59]. Indeed, we showed here that SIRT6 can increase glycolysis in NSCLC cells by promoting its downstream HIF-1α/HK2 pathway to induce cell resistance to erlotinib. Similar to our findings, one literature found that glycolysis inhibition sensitizes non-small cell lung cancer with T790M mutation to irreversible EGFR inhibitors [14]. And another study found that reducing glucose uptake and intracellular ATP level contributes to the antiproliferative activity of EGFR-TKI in EGFR wild-type NSCLC cells and EGFR sensitive mutant cells [60]. These findings suggest that glycolysis is an important drug resistance mechanism in a variety of EGFR-mutant NSCLC. Therefore, we speculate that SIRT6 may be involved in TKI resistance in a variety of EGFR mutant cells (including T790M, L858R, and 19del), which will be confirmed in our future studies.

Currently, given that SIRT6 plays a central role in several processes, such as DNA repair, metabolism, and tumorigenesis, some SIRT6 modulators have been developed as potential weapons for treatment of diseases [61]. Quinazolinedione inhibits SIRT6 via competition with the peptide substrate, and sensitizes pancreatic cancer cells to gemcitabine and to olaparib [62]. In addition, OSS-128167 is a novel SIRT6 inhibitor and presents excellent anti-lymphoma effects by inhibiting PI3K/Akt/mTOR pathway [34]. JYQ-42, as a selective and non-competitive inhibitor of SIRT6, can inhibit cell migration and cytokine production in pancreatic cancer cells, showing potent anti-cancer effects [36]. Conversely, some researchers maintain that the SIRT6 allosteric activator can inhibit the proliferation of cancer cells. The researchers found that MDL-800 inhibits the proliferation of HCC cells via SIRT6-driven cell-cycle arrest, and they also suggested that MDL-800 suppresses proliferation and enhances osimertinib therapy in NSCLC [37, 63].

Why are these studies inconsistent? First, the function of SIRT6 is complex. In different tumor types and processes of tumorigenesis, and even in the same tumor under different circumstances, its functions are often two-sided, resulting in inconsistent findings in several studies [64]. For lung cancer, especially NSCLC, there are reports supporting our research: SIRT6 is highly expressed in NSCLC, and SIRT6 is associated with poor prognosis of NSCLC patients [54, 65]. In addition, inhibiting SIRT6 can decrease cisplatin (DDP) resistance in NSCLC cells [66]. In particular, a recent meta-analysis involving 3 solid tumors found that reduced SIRT6 expression was shown to be associated with improved OS [67]. Second, the research methods and models are different, which may also lead to different results. Different inhibitors or activators used in the experiments may affect the experimental results. There are many SIRT6 modulators, and their functional targets are different, which may also lead to the inconsistent experimental results [61]. A recent study concluded that SIRT6 activator MDL-800 could inhibit NSCLC proliferation and enhance the EGFR-TKIs effect. In this study, MDL-800 was used to enhance the deacetylation activity of SIRT6, but another study showed that the deacetylase-independent function of SIRT6 promoted the expression of anti-apoptotic genes through the transcription factor GATA4 to prevent doxorubicin toxicity [68]. However, we used lentivirus transfection technology to directly interfere with SIRT6 gene expression, which may also lead to inconsistent results. Moreover, the different cell models used in the experiment will also have an impact on the experimental results. MDL-800 acts on osimertinib-resistant cells, while we used Erlotinib-resistant cells. There is a large difference in gene expression between these cell lines, which may also be one of the reasons for the different findings. Therefore, it is necessary to further study the function of SIRT6, which also indicates that there is a long way to go to develop effective and reliable molecule modulators for clinical use.

Our data suggest that SIRT6 regulates the metabolism of NSCLC cells and renders NSCLC cells resistant to erlotinib by promoting glycolysis. However, the study has some limitations. The metabolism of sugar, fat and amino acids plays an important role in the life process of organisms. Therefore, the influence of the metabolism of other compounds (such as lipids) on the sensitivity of erlotinib should also be studied, which may provide us with additional new effective intervention targets. Whether SIRT6 can regulate erlotinib resistance by reprogramming lipid metabolism is still unclear. In addition, our study was only conducted in the erlotinib, and the role of SIRT6 in the other TKIs needs to be verified by further experiments, and we have successfully induced osimertinib-resistant cells, and the potential role of SIRT6 in the third generation of TKIs will be further studied in the future. More importantly, if SIRT6 inhibitors are developed for clinical use, we also need to consider their long-term side effects.

In summary, SIRT6 knockdown can significantly strengthen the inhibitory effect of erlotinib on drug-resistant cells, improve the sensitivity of drug-resistant cells to erlotinib, and promote the apoptosis of drug-resistant cells. Therefore, SIRT6 can be an effective new target for the treatment of EGFR-TKI resistance in NSCLC patients. SIRT6 inhibition alone or combined with EGFR-TKIs may be a new strategy to address the emergence of resistance after EGFR-TKI treatment.

## Data Availability

The datasets used or analyzed during the current study are available from the corresponding author on reasonable request.
